# Feedback Regulation of *O*-GlcNAc Transferase through Translation Control to Maintain Intracellular *O*-GlcNAc Homeostasis

**DOI:** 10.3390/ijms22073463

**Published:** 2021-03-27

**Authors:** Chia-Hung Lin, Chen-Chung Liao, Mei-Yu Chen, Teh-Ying Chou

**Affiliations:** 1 Division of Molecular Pathology, Department of Pathology and Laboratory Medicine, Taipei Veterans General Hospital, Taipei 11217, Taiwan; chiahunglin0222@gmail.com; 2 Metabolomics-Proteomics Research Center, National Yang-Ming University, Taipei 11221, Taiwan; ccliao@ym.edu.tw; 3 Metabolomics-Proteomics Research Center, National Yang Ming Chiao Tung University, Hsinchu 30010, Taiwan; 4 Faculty of Medicine, School of Medicine, National Yang-Ming University, Taipei 11221, Taiwan; 5 Faculty of Medicine, School of Medicine, National Yang Ming Chiao Tung University, Hsinchu 30010, Taiwan; 6 Institute of Biochemistry and Molecular Biology, National Yang-Ming University, Taipei 11221, Taiwan; 7 Institute of Biochemistry and Molecular Biology, National Yang Ming Chiao Tung University, Hsinchu 30010, Taiwan; 8 Cancer Progression Research Center, National Yang-Ming University, Taipei 11221, Taiwan; 9 Cancer Progression Research Center, National Yang Ming Chiao Tung University, Hsinchu 30010, Taiwan; 10 Institute of Clinical Medicine, National Yang-Ming University, Taipei 11221, Taiwan; 11 Institute of Clinical Medicine, National Yang Ming Chiao Tung University, Hsinchu 30010, Taiwan

**Keywords:** epigenetics, eukaryotic translation initiation factor 4E-binding protein 1 (EIF4EBP1), histone deacetylase (HDAC), *O*-GlcNAcase (OGA), *O*-GlcNAcylation, *O*-linked *N*-acetylglucosamine (*O*-GlcNAc), *O*-GlcNAc homeostasis, *O*-GlcNAc transferase (OGT), post-translational modification, translation control

## Abstract

Protein *O*-GlcNAcylation is a dynamic post-translational modification involving the attachment of *N*-acetylglucosamine (GlcNAc) to the hydroxyl groups of Ser/Thr residues on numerous nucleocytoplasmic proteins. Two enzymes are responsible for *O*-GlcNAc cycling on substrate proteins: *O*-GlcNAc transferase (OGT) catalyzes the addition while *O*-GlcNAcase (OGA) helps the removal of GlcNAc. *O*-GlcNAcylation modifies protein functions; therefore, dysregulation of *O*-GlcNAcylation affects cell physiology and contributes to pathogenesis. To maintain homeostasis of cellular *O*-GlcNAcylation, there exists feedback regulation of OGT and OGA expression responding to fluctuations of *O*-GlcNAc levels; yet, little is known about the molecular mechanisms involved. In this study, we investigated the *O*-GlcNAc-feedback regulation of OGT and OGA expression in lung cancer cells. Results suggest that, upon alterations in *O*-GlcNAcylation, the regulation of OGA expression occurs at the mRNA level and likely involves epigenetic mechanisms, while modulation of OGT expression is through translation control. Further analyses revealed that the eukaryotic translation initiation factor 4E-binding protein 1 (4E-BP1) contributes to the downregulation of OGT induced by hyper-*O*-GlcNAcylation; the S5A/S6A *O*-GlcNAcylation-site mutant of 4E-BP1 cannot support this regulation, suggesting an important role of *O*-GlcNAcylation. The results provide additional insight into the molecular mechanisms through which cells may fine-tune intracellular *O*-GlcNAc levels to maintain homeostasis.

## 1. Introduction

*O*-GlcNAcylation is a highly inducible, reversible and abundant post-translational modification of proteins [1]. This glycosylation is catalyzed by the enzyme *O*-GlcNAc transferase (OGT) [2,3], which transfers the *N*-acetylglucosamine moiety from uridine 5′-diphospho-*N*-acetylglucosamine (UDP-GlcNAc) to serine or threonine residues on the target proteins. Another enzyme, *O*-GlcNAcase (OGA), removes *O*-GlcNAc from proteins [4,5]. Distinct from the complex polyglycan modification on cell surface proteins, *O*-GlcNAc, a monosaccharide attached through a β-linkage, is a major form of glycosylation found on cytoplasmic and nuclear proteins. Unlike dynamic protein phosphorylation which involves many different kinases and phosphatases, *O*-GlcNAcylation of numerous proteins is regulated by one single OGT and a sole OGA in cells.

Since UDP-GlcNAc is synthesized through the hexosamine biosynthetic pathway (HBP), *O*-GlcNAcylation is sensitive to the levels of nutrients in the cellular environment [6]. Alterations in nutrient status, hormone levels, or extracellular environmental stress can result in changes to the total level of *O*-GlcNAc on proteins [7,8]. *O*-GlcNAcylation participates in many crucial cellular functions, including transcription, translation, epigenetics, cellular signaling and metabolism [9,10]. Therefore, maintenance of *O*-GlcNAc homeostasis is very important for proper cellular functions. Dysregulation of *O*-GlcNAcylation perturbs cell physiology and may contribute to the etiology of various diseases, such as diabetes, neurodegenerative disorders and cancer [11].

Studies have shown that OGT and OGA expression are sensitive to fluctuations in cellular *O*-GlcNAc levels. For instance, downregulation of OGT protein levels under pharmacological inhibition of OGA has been demonstrated in a range of cell lines including those of cervical cancer, neuroblastoma, leukemia, colon cancer, and fibroblast origins [12,13,14,15]. Conversely, OGA protein levels are downregulated upon OGT inhibition, OGT knockdown, or OGT knockout in cultured mammalian ovary or kidney cell lines [16,17,18]. These findings suggest that cells coordinate the expression of OGT and OGA to buffer them from drastic shifts in *O*-GlcNAcylation levels. However, the mechanisms for coordinating OGT and OGA expression remain elusive. A few reports have indicated that transcriptional control is involved in regulating OGA and/or OGT expression for *O*-GlcNAc homeostasis [13,15,19]. There is also a regulatory intron retention in the *OGT* transcript discovered to be responsive to cellular *O*-GlcNAc levels [14]. With the sparse information, it is unclear whether all cell types utilize conserved mechanisms to regulate the expression of OGA and OGT upon changes of cellular *O*-GlcNAcylation. We have previously revealed a positive correlation between OGT and OGA protein expression in lung adenocarcinoma tissues [20]. In this study, we further investigated the regulatory loops involved in adjusting the expression of the two *O*-GlcNAc-cycling enzymes when changes of cellular *O*-GlcNAcylation status occur in lung cancer cells. We provide evidence supporting that OGA regulation occurs at the transcript level while OGT is under translation control in response to changes in cellular *O*-GlcNAc levels. Our data also revealed a role of the eukaryotic translation initiation factor 4E-binding protein 1 (4E-BP1) in *O*-GlcNAc-feedback modulation of OGT protein level.

## 2. Results

### 2.1. Changes in O-GlcNAc Levels Lead to Feedback Regulation of OGT and OGA Protein Expression in Lung Cancer Cells

**Figure 1:**
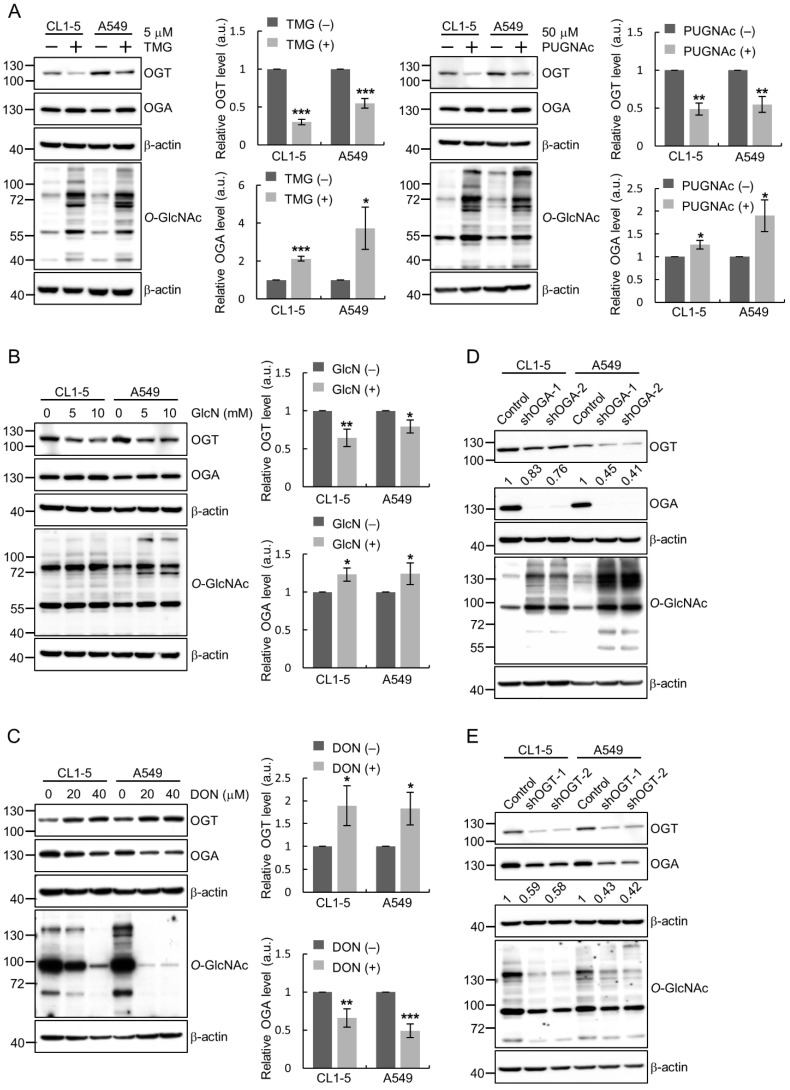
Changes of *O*-GlcNAc transferase (OGT) and *O*-GlcNAcase (OGA) protein expression in response to alteration of *O*-GlcNAc levels in lung cancer cells. (**A**–**C**) Cells were subjected to indicated treatments for 24 h and total lysates were examined for OGT, OGA and *O*-GlcNAc levels by Western blotting analysis; β-actin, loading control. Signal intensities were quantitated using ImageJ software; means ± SD from multiple independent experiments are shown. *, *p* < 0.05; **, *p* < 0.01; ***, *p* < 0.001. (**A**) Elevation of *O*-GlcNAcylation by OGA inhibition using Thiamet G (TMG) or PUGNAc. (**B**) Upregulation of *O*-GlcNAc levels by glucosamine (GlcN) treatment to activate the hexosamine biosynthetic pathway (HBP). Quantitative results from 10 mM treatment are shown. (**C**) Downregulation of *O*-GlcNAc levels following the inhibition of HBP by the glutamine fructose-6-phosphate amidotransferase (GFAT) inhibitor 6-Diazo-5-oxo-L-norleucine (DON). Quantitative results from 40 μM treatment are shown. (**D**–**E**) CL1-5 and A549 cells were infected with shRNA-expressing lentiviruses to knockdown OGA (**D**) or OGT (**E**) expression. Total lysates from infected cells were examined for levels of OGT, OGA and *O*-GlcNAc by Western blotting analysis. Normalized OGT/β-actin (**D**) and OGA/β-actin (**E**) signal ratios are shown under the blots.

To investigate the existence of feedback loops for the maintenance of cellular *O*-GlcNAc homeostasis in lung cancer cells, we examined the expression of OGA and OGT under conditions with varying extents of cellular *O*-GlcNAcylation. When cultured CL1-5 or A549 human lung adenocarcinoma cells were treated with an OGA inhibitor, either Thiamet G (TMG) or PUGNAc, to elevate *O*-GlcNAcylation, the amount of OGT protein decreased while that of OGA increased (Figure 1A). When cells were treated with glucosamine (GlcN) which activates the flux of the HBP, cellular *O*-GlcNAcylation levels were transiently increased (Figure S1); this treatment resulted in increased OGA and decreased OGT protein levels (Figure 1B). Conversely, treating cells with the glutamine fructose-6-phosphate amidotransferase (GFAT) inhibitor 6-Diazo-5-oxo-L-norleucine (DON), which inhibits the HBP and thereby reduces intracellular UDP-GlcNAc levels, led to the up-regulation of OGT and the down-regulation of OGA (Figure 1C). Additionally, we manipulated *O*-GlcNAc levels by silencing the endogenous expression of OGA or OGT using shRNA-expressing lentiviruses. Knockdown of OGA with two different shRNAs in CL1-5 and A549 cells resulted in increased *O*-GlcNAc levels and reduced OGT expression as compared to those of the control cells (Figure 1D), while silencing of OGT expression decreased *O*-GlcNAc levels and lowered OGA expression (Figure 1E). Collectively, we observed that manipulating the cellular *O*-GlcNAcylation status, either by pharmacological treatments or molecular genetic approaches, affected the protein levels of OGT and OGA in a direction towards maintaining *O*-GlcNAc homeostasis. This suggests a possibility that the expression of these two enzymes is modulated by intracellular *O*-GlcNAc levels through feedback mechanisms in lung cancer cells.

### 2.2. Regulation of OGA Expression in Response to Changes in Cellular O-GlcNAcylation Occurs at the mRNA Level and Likely Involves Epigenetic Mechanisms

**Figure 2:**
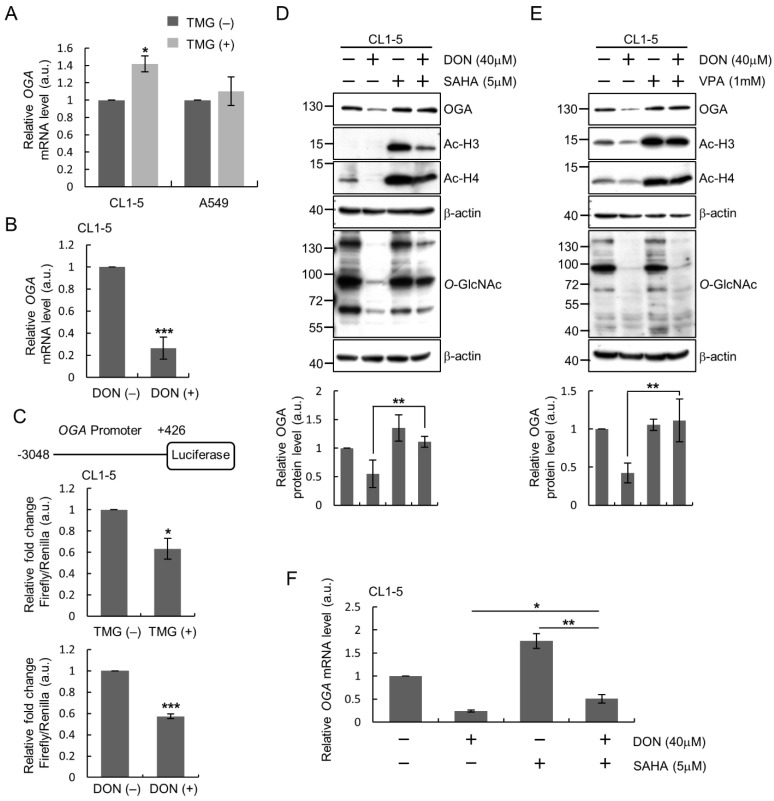
Regulation of OGA expression at the transcript level in response to alteration of *O*-GlcNAcylation in lung cancer cells. (**A**,**B**) Analysis of endogenous *OGA* mRNA levels. CL1-5 or A549 cells were treated for 24 h with TMG (5 μM) to elevate *O*-GlcNAcylation (**A**) or DON (40 μM) to downregulate *O*-GlcNAc level (**B**) and RNA preparation for RT-qPCR analysis. (**C**) Luciferase assay for *OGA* promoter activity. The *OGA* promoter (−3048~+426) was cloned into the pGL3-basic vector upstream of the firefly luciferase reporter gene. CL1-5 cells were transfected with pGL3-basic or the *OGA* promoter reporter plasmid and treated with or without TMG or DON before assays. (**D**,**E**) Effect of inhibiting histone deacetylases (HDACs) on regulation of OGA protein levels in DON-treated CL1-5 cells. CL1-5 cells were treated with or without DON (40 μM) and/or SAHA (5 μM) (**D**) or VPA (1 mM) (**E**) for 24 h. Levels of OGA, Ac-H3, Ac-H4 and *O*-GlcNAc in the total cell lysates were examined by Western blotting analysis. (**F**) Effect of HDAC inhibition on endogenous *OGA* mRNA levels. Cells were treated as indicated for 24 h and subjected to RT-qPCR analysis. Shown in all the bar graphs are means ± SD from multiple independent experiments. *, *p* < 0.05; **, *p* < 0.01; ***, *p* < 0.001.

We set out to determine at which level of gene expression that regulation of OGA might occur in lung cancer cells in response to changes of *O*-GlcNAcylation status. We compared the *OGA* transcript levels in cells before and after perturbing cellular *O*-GlcNAcylation. RT-qPCR results revealed that in CL1-5 cells treated with the OGA inhibitor TMG to increase *O*-GlcNAc levels, *OGA* mRNA expression was significantly increased as compared to that of untreated cells (Figure 2A), although the changes of *OGA* mRNA level in A549 cells did not reach statistical significance. On the other hand, when CL1-5 cells were treated with DON to inhibit the HBP activity and reduce cellular *O*-GlcNAc level, the endogenous *OGA* transcript level was significantly lowered as compared to that of untreated cells (Figure 2B).

To explore the possibility of transcriptional control, we performed luciferase reporter assays to assess the activity of *OGA* promoter after manipulation of *O*-GlcNAcylation. Intriguingly, the changes in *OGA* promoter activity after pharmacological manipulation of *O*-GlcNAcylation did not totally parallel the changes in endogenous OGA transcript or protein levels: we observed decreased promoter activity in CL1-5 cells treated with either TMG or DON as compared to that in the untreated cells (Figure 2C). Considering that the luciferase reporter assay was performed using exogenous plasmid DNA for transient transfection of cells, the discrepancy prompted us to examine whether the underlying mechanism of *O*-GlcNAc-stimulated OGA upregulation involves epigenetics. Many previous studies have demonstrated that *O*-GlcNAcylation can regulate chromatin remodeling to control gene expression [21,22,23,24]. To test the involvement of histone modifications, we treated CL1-5 cells with a histone deacetylase (HDAC) inhibitor, either suberoylanilide hydroxamic acid (SAHA) or valproic acid (VPA), to increase histone acetylation, and examined the effect on OGA expression after manipulation of *O*-GlcNAcylation; results demonstrated that DON-induced OGA protein downregulation was reversed by HDAC inhibitors (Figure 2D,E). Results of RT-qPCR analysis also showed that HDAC inhibition elevated endogenous *OGA* transcript levels whether cells were treated with DON or not, and the DON-induced downregulation of *OGA* mRNA levels was slightly blunted by SAHA treatment (Figure 2F).

Taken together, these results suggest that OGA regulation in response to altered cellular *O*-GlcNAcylation occurs at the transcript level and may happen, at least in part, through epigenetic modulation.

### 2.3. Regulation of OGT Expression in Response to Changes in Cellular O-GlcNAcylation Involves Translation Control

**Figure 3:**
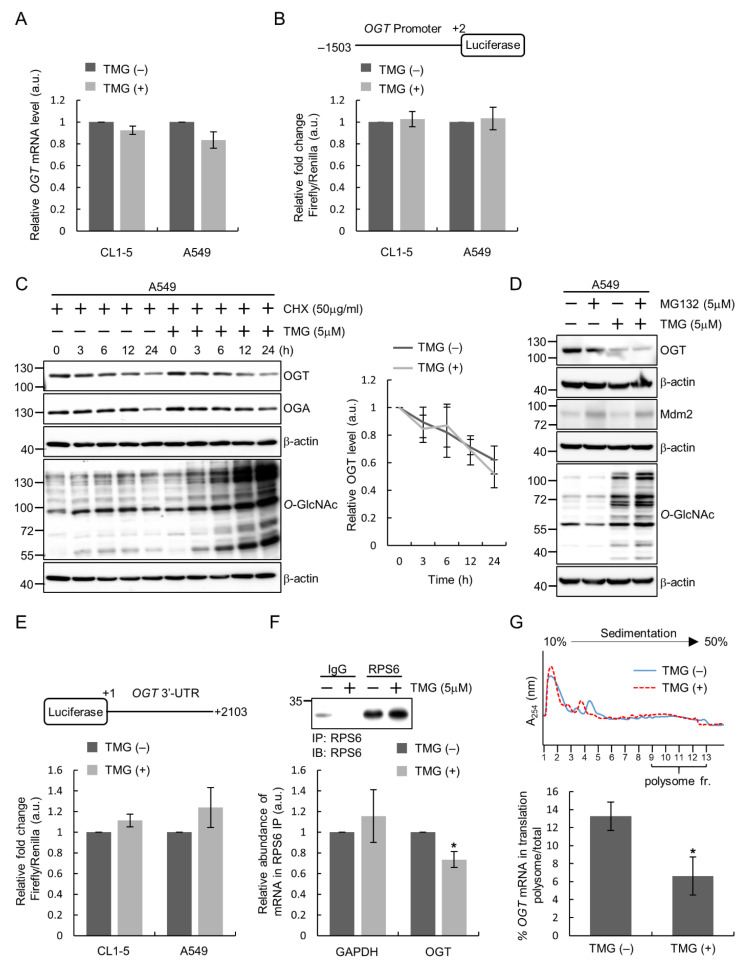
Translation control of OGT expression in response to alteration of *O*-GlcNAcylation in lung cancer cells. (**A**) Lung cancer cells were treated or not with TMG (5 μM) for 24 h and analyzed for *OGT* mRNA expression by RT-qPCR. (**B**) The *OGT* promoter (−1503~+2) was cloned into the pGL3-basic vector upstream of the firefly luciferase reporter gene. Lysates from cells transfected with pGL3-basic or the *OGT* promoter reporter plasmid treated with or without TMG (5 μM) for 24 h were assayed for luciferase activity. (**C**) Lysates from cells treated with/without TMG (in the presence of cycloheximide (CHX) to inhibit translation) for indicated time were analyzed by Western blotting analysis. Signal intensities were quantitated using ImageJ software; relative OGT levels are OGT/β-actin signal ratios normalized to the time 1 value, and means ± SD from multiple experiments are shown. (**D**) Cells were treated or not with TMG and/or MG132 as indicated for 24 h and lysates were subjected to Western blotting analysis. Mdm2 was a positive control for MG132 treatment. (**E**) The 3′-UTR of *OGT* (1~2103) was cloned into pGL3-control downstream of the firefly luciferase reporter gene. Lung cancer cells were transfected with pGL3-control or the *OGT*-3′UTR reporter plasmid and treated with or without TMG (5 μM) for 24 h before lysates were prepared for luciferase assays. (**F**) CL1-5 cells were treated with or without TMG (5 μM) for 24 h. RPS6-immunoprecipitation (IP) was performed to pulldown the ribosome–mRNA complex. Immunoprecipitates were examined for RPS6 by Western blotting analysis. Levels of *OGT* and *GAPDH* mRNAs in RPS6-IP were quantified using RT-qPCR. (**G**) Lysates from CL1-5 cells treated or not with TMG (5 μM) were subjected to sucrose gradient fractionation for ribosome profiling. The amounts of polysome-associated *OGT* transcript (in fractions 9–13) were quantified using RT-qPCR. *, *p* < 0.05.

To explore the regulation of OGT expression responding to perturbation of cellular *O*-GlcNAcylation, we first performed RT-qPCR analysis. Results revealed that TMG treatment did not induce changes in endogenous *OGT* mRNA levels in lung cancer cell lines (Figure 3A), suggesting that the aforementioned decrease in the amount of OGT protein after TMG treatment is regulated at a post-transcriptional level. In line with this, when the activity of *OGT* promoter was examined using luciferase reporter assays, TMG-treated cells showed similar levels of promoter activity as compared to that of untreated cells (Figure 3B). We next examined the protein stability of OGT in response to changes in *O*-GlcNAc levels by following the changes of OGT protein level after adding cycloheximide (CHX) to inhibit de novo protein synthesis. Results showed that the half-life of OGT protein in TMG-treated cells was similar to that in vehicle-treated control cells (Figure 3C). Additionally, inhibition of the proteasome degradation pathway by MG132 did not restore the TMG-induced decrease in OGT protein amount (Figure 3D). These results suggest that the regulation of OGT is not through a post-translational mechanism such as controlling protein degradation. We turned to ask if the post-transcript level regulation of OGT expression by *O*-GlcNAc levels involves trans-regulatory factors, such as RNA-binding proteins or miRNAs, which act on the 3′ untranslated region (3′UTR) of the *OGT* transcript. Results from luciferase activity assays using an *OGT*-3′UTR luciferase reporter showed that TMG treatment did not lead to significant changes in the luciferase activity from *OGT*-3′UTR (Figure 3E), excluding the possibility of 3′UTR-mediated regulation of OGT expression.

Given the above results, we next investigated whether OGT regulation is through the control of protein translation. A ribosome pulldown was performed by immunoprecipitating the 40S ribosomal protein S6 (RPS6), and ribosome-bound mRNAs were subsequently extracted and analyzed for *OGT* mRNA level by RT-qPCR. Results showed that in TMG-treated cells, the amount of ribosome-bound *OGT* mRNA was decreased as compared to that in control cells, while the *GAPDH* mRNA level displayed no significant changes after TMG treatment (Figure 3F). To confirm that manipulating cellular *O*-GlcNAc levels by TMG treatment affects the amount of *OGT* mRNA being translated, we conducted a 10–50% sucrose gradient fractionation and examined the distribution of *OGT* mRNA in fractions containing polysomes. We found that the percentage of *OGT* mRNA in the polysomal fractions decreased from ~13% in cells with no TMG treatment to ~7% in TMG-treated cells (Figure 3G), indicating that the translational efficiency of *OGT* mRNA was reduced by elevated *O*-GlcNAcylation. Taken together, these results support the notion that elevation of cellular *O*-GlcNAc levels negatively regulates OGT protein expression by suppressing *OGT* mRNA translation.

### 2.4. PI3K Can Regulate OGT Expression and 4E-BP1 Contributes to the Modulation of OGT Level in Response to Changes in Cellular O-GlcNAcylation

**Figure 4:**
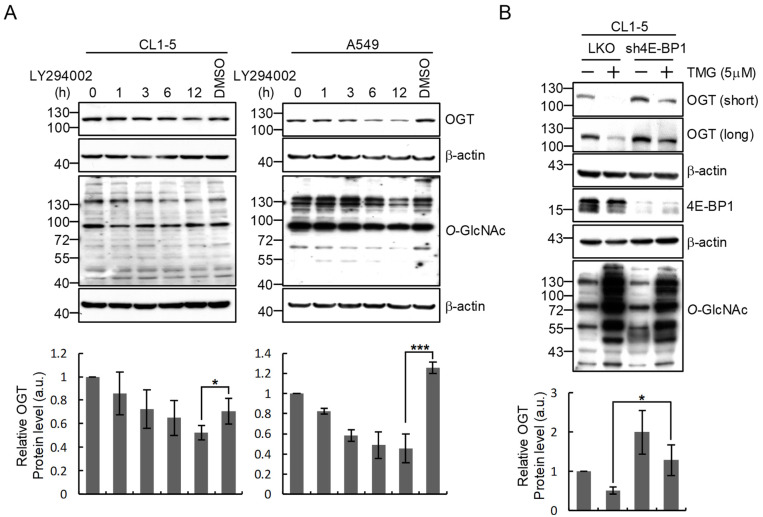
PI3K can regulate OGT expression and 4E-BP1 contributes to the modulation of OGT level in response to changes in cellular *O*-GlcNAcylation in lung cancer cells. (**A**) Inhibition of the PI3K signaling pathway decreases OGT protein expression. CL1-5 and A549 cells were treated with LY294002 (20 μM), a PI3K inhibitor, for the indicated lengths of time. Levels of OGT and *O*-GlcNAc were examined by Western blotting analysis. (**B**) Silencing of 4E-BP1 expression in CL1-5 cells rescues the decrease in OGT protein level after TMG treatment. 4E-BP1-silenced (sh4E-BP1) CL1-5 cells were treated with TMG (5 μM) for 24 h. Levels of OGT, 4E-BP1 and *O*-GlcNAc in total cell lysates were examined by Western blotting analysis. Western blotting signals were quantitated using ImageJ software and relative OGT/β-actin signal ratios were calculated; means ± SD from multiple experiments are shown. *, *p* < 0.05; ***, *p* < 0.001.

We further explored the underlying mechanism for the translation control of OGT expression. Since protein translational efficiency is tightly regulated by the critical PI3K/mTORC1/4E-BP1 signaling pathway that controls cell growth, proliferation and survival [25], we tested if this signaling pathway is involved in OGT regulation by employing a strong inhibitor of PI3K, i.e., LY294002, to inhibit the pathway activity and check the effect on OGT levels. Results showed a time-dependent decrease in OGT protein amount in LY294002-treated cells to a level significantly lower than that in DMSO-treated cells (Figure 4A), suggesting a role of the PI3K-linked pathway in modulating OGT expression. To address the role of this pathway in *O*-GlcNAc-feedback regulation of OGT expression, we carried out shRNA-mediated silencing of the downstream translational repressor 4E-BP1 and examined the expression of OGT in response to *O*-GlcNAcylation elevation by TMG treatment. We found that 4E-BP1 knockdown resulted in a rescue of the TMG/hyper-*O*-GlcNAcylation-induced decrease in OGT protein levels compared to that in the control cells (Figure 4B). These results support that 4E-BP1 may contribute to the feedback regulation of OGT expression by elevated cellular *O*-GlcNAcylation.

### 2.5. 4E-BP1 Is O-GlcNAcylated on Ser5 and Ser6 and Mutations of These Sites Abolishes the Contribution of 4E-BP1 to OGT Regulation in Response to Changes in Cellular O-GlcNAcylation

**Figure 5:**
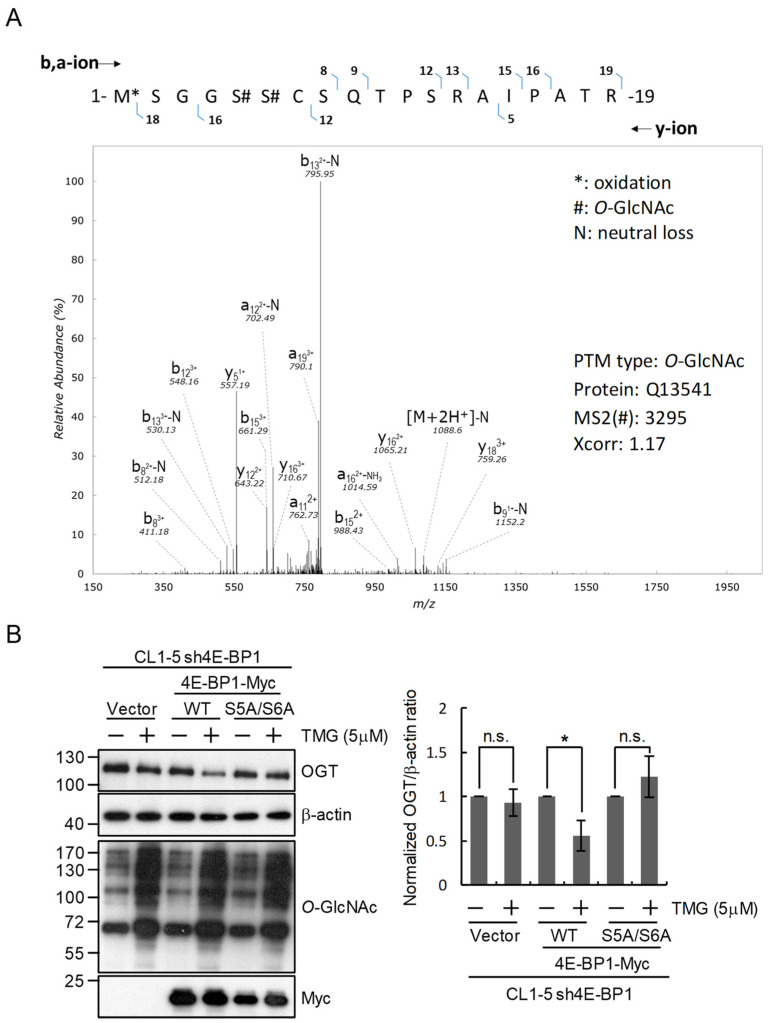
Mutation of 4E-BP1 *O*-GlcNAcylation sites abolishes its contribution to the regulation of OGT expression in response to changes of cellular *O*-GlcNAcylation in lung cancer cells. (**A**) Tandem mass spectrum of the *O*-GlcNAcylated 4E-BP1 peptide with the sequence 1-MSGGSSCSQTPSRAIPATR-19. (**B**) Effect of mutating 4E-BP1 *O*-GlcNAcylation sites on OGT regulation. 4E-BP1-silenced (sh4E-BP1) CL1-5 cells were transfected with the control vector or a plasmid expressing wild-type (WT) or mutant 4E-BP1 with Ser to Ala substitutions on the *O*-GlcNAcylation sites Ser-5 and Ser-6 (S5A/S6A). The resulting transfected cells were treated or not with TMG (5 μM) for 24 h. Levels of OGT, 4E-BP1 and *O*-GlcNAc in total cell lysates were examined by Western blotting analysis. Signal intensities were quantitated using ImageJ software to calculate the normalized OGT/β-actin signal ratios; means ± SD from multiple experiments are shown. *, *p* < 0.05; n.s., not statistically significant.

We next inquired how 4E-BP1 may mediate the *O*-GlcNAc-feedback regulation of OGT expression. Since it has been reported that 4E-BP1 is *O*-GlcNAcylated [26], a plausible scenario is that 4E-BP1 serves as a sensor of cellular *O*-GlcNAcylation status. To test this hypothesis, we may mutate the *O*-GlcNAcylation sites in 4E-BP1 to disrupt its ability to sense cellular *O*-GlcNAcylation levels. Therefore, we first attempted the identification of *O*-GlcNAcylated sites on 4E-BP1 by immunoprecipitation and subsequent liquid chromatography and tandem mass spectrometry analysis. Results revealed the *O*-GlcNAcylation status on Ser5 and Ser6 of 4E-BP1 (Figure 5A). We next generated a substitution mutant of 4E-BP1 by replacing these two Ser residues with Ala and tested the function of mutant 4E-BP1 in supporting the *O*-GlcNAc-feedback regulation of OGT expression. Results showed that while expressing wild-type 4E-BP1 in 4E-BP1-knockdown cells restored the TMG-induced downregulation of OGT protein level, the S5A/S6A mutant 4E-BP1 was not able to support this *O*-GlcNAc-feedback regulation of OGT expression (Figure 5B). These results are consistent with the notion that 4E-BP1 serves as a cellular *O*-GlcNAcylation sensor for coordinating the regulation of OGT towards maintaining *O*-GlcNAc homeostasis.

## 3. Discussion

Our investigation of the regulation of *O*-GlcNAc-cycling enzymes in lung cancer cells in response to changes of *O*-GlcNAcylation status corroborated a general phenomenon observed in other cell types and systems [12,13,14,15,16,17,18]: cellular *O*-GlcNAcylation needs to be maintained at a homeostatic level, presumably to preserve proper cell functions, and this is achieved by compensatory modulation of the expression of OGT and OGA. Aberrant *O*-GlcNAcylation status has been linked to various human disorders, such as diabetes, cancer, neurodegenerative and cardiovascular disease, and targeting *O*-GlcNAcylation is emerging as a novel direction for developing therapeutics [11,27]. Therefore, understanding the mechanisms by which cells sense fluctuations in homeostatic levels of *O*-GlcNAc and regulate OGT and OGA expression is very important.

In this study, we have demonstrated that the regulatory mechanisms associated with modulating levels of *O*-GlcNAc-cycling enzymes according to cellular *O*-GlcNAcylation status appear to be different for OGA and OGT in lung cancer cells. Consistent with previous studies [13,15], we found that *OGA* transcript levels exhibited compensatory changes responding to perturbation of cellular *O*-GlcNAcylation status. A study using cDNA microarray analysis also showed that the gene encoding OGA is markedly down-regulated by silencing of *OGT* expression in prostate cancer cell lines [28]. Together, these findings suggest that *OGA* regulation at the transcript level is a general modulatory mechanism happening in diverse cell types. Interestingly, when investigating the transcriptional control of the *OGA* promoter using luciferase reporter assays, we did not obtain results consistent with the effects on OGA protein levels, indicating that there is some other regulatory mechanism involved in modulating the *OGA* transcript in response to *O*-GlcNAc levels.

Many studies have demonstrated that *O*-GlcNAcylation participates in epigenetics by modifying histone proteins, including H2A, H2B, H3 and H4 [29,30,31,32,33]. H2B *O*-GlcNAcylation at Ser-112 is associated with transcribed genes, including the OGA-encoding gene [30]. Ten-eleven translocation methylcytosine dioxygenase (TET) proteins can interact with OGT to facilitate its recruitment to chromatins and to promote histone *O*-GlcNAcyaltion [34,35,36]. Our results showed that inhibition of HDAC could counteract the DON-induced decrease in OGA protein, suggesting that histone acetylation may collaborate with *O*-GlcNAcylation in regulating *OGA* gene transcription following changes in *O*-GlcNAc levels. The detailed mechanism underlying this epigenetic regulation awaits further elucidation.

On the other hand, our data clearly demonstrated that modulation of OGT expression in response to changes of *O*-GlcNAcylation is conducted through translation control in lung cancer cells. Following analysis of a ribosome pulldown assay and polysome profiling, we found that elevation of cellular *O*-GlcNAcylation reduces the amount of *OGT* transcript associated with the active translational machinery, leading to decreased translational efficiency to produce OGT protein. Our findings are consistent with some reports which suggest post-transcriptional mechanisms of *OGT* regulation, for example, in SH-SY5Y, HeLa, K562 and HCT116 cells [13,15]. However, reports showing *OGT* regulation at the transcript level also exist in the literature. A study has demonstrated that overexpression of *OGA* in primary hepatocytes or HEK293T cells can increase *OGT* transcription through CCAAT/enhancer-binding protein β [37]. Additionally, negative regulation of *OGT* transcription by the transcription factor E2F1 has been shown in mouse fibroblasts and HEK293 cells [19]. As these seemingly discrepant findings for the mechanisms of *OGT* regulation were obtained in different types of cells, it remains to be clarified whether *OGT* expression is regulated in a cell-type-specific manner. Intriguingly, we noted that studies supporting post-transcriptional regulation of *OGT* expression have used cancer cells in experiments, while those demonstrating *OGT* regulation at the transcript level have been done in non-tumorous cells; our results obtained in lung cancer cells seem to be consistent with this notion. It is conceivable that homeostatic mechanisms for maintaining stable *O*-GlcNAcylation status in cancer and non-cancer cells are different. Obviously, further investigations are required to elucidate if cancer and non-cancer cells utilize differential mechanisms to regulate *OGT* expression for maintaining cellular *O*-GlcNAc homeostasis.

Emerging evidence has pointed out another mechanism involving the control of intron retention in regulating the expression of OGT and OGA. It has been shown in both non-tumor (293A-TOA) and cancer (HCT116) cells that TMG treatment induces intron retention in the *OGT* transcript to regulate OGT expression; the intron retention is responsive to changes in *O*-GlcNAc levels and is regulated by a conserved intronic splicing silencer [14]. A very recent report describes that cellular *O*-GlcNAc levels affect the splicing of detained introns in both *OGT* and *OGA* transcripts to control the amounts of mRNAs in HEK293T and HCT116 cells [38]. In this study, we did not investigate intron retention of *OGT* and *OGA* transcripts in our cell line models. It remains to be determined whether this control mechanism of OGT and OGA expression is also employed by lung cancer cells.

Previous studies have indicated that the mTOR signaling pathway plays a vital role in regulating protein stability and translation initiation. In breast cancer cells, the PI3K–mTOR–MYC signaling pathway is required for elevation of OGT and *O*-GlcNAcylation [39]. In hepatic and colon cancer cells, pharmacological inhibition of mTOR activity by rapamycin decreases OGT expression by regulating protein stability [40,41]. However, our results did not show changes of OGT protein stability by TMG treatment. It has been demonstrated that hyperglycemia-induced *O*-GlcNAcylation of 4E-BP1, a downstream target of mTORC1, regulates protein translation in diabetes pathogenesis [26,42,43]. *O*-GlcNAcylation of 4E-BP1 enhances its binding to eukaryotic translation initiation factor 4E (eIF4E) and inhibits cap-dependent translation [43]. Therefore, it is expected that the levels of a plethora of proteins would be affected by *O*-GlcNAcylation in a 4E-BP1-dependent manner. Examples of such proteins include superoxide dismutase 2 (SOD2) and Cd40 [44,45]. In mouse retina, TMG treatment inhibits the translation of SOD2 in a 4E-BP-dependent manner; the functional significance of this regulation is evidenced by the increased oxidative stress in the retina of diabetic mice which may be attributed to altered translation of SOD [45]. Conversely, because Cd40 mRNA undergoes cap-independent translation, TMG treatment causes an increase in association of Cd40 mRNA with ribosomes and enhancement of Cd40 translation in Muller glia cells in mouse retina [44]. Our study establishes OGT as another example regulated by *O*-GlcNAcylation in a 4E-BP1-dependent way. Results showing that silencing of 4E-BP1 expression rescues the hyper-*O*-GlcNAcylation-induced decrease in OGT protein have highlighted a functional significance of 4E-BP1 in the mechanisms for maintaining cellular *O*-GlcNAcylation homeostasis. To our knowledge, this is the first example showing a functional link between 4E-BP1 and OGT regulation.

Our results of immunoprecipitation coupled with mass spectrometry analysis revealed Ser5 and Ser6 as *O*-GlcNAcylation sites on 4E-BP1. By mutating these two sites and showing that the S5A/S6A 4E-BP1 mutant protein no longer supports the hyper-*O*-GlcNAcylation-induced downregulation of OGT protein level like the wild-type 4E-BP1 protein does, we provide evidence supporting a scenario in which the *O*-GlcNAcylation of proteins regulating translation may represent one key mechanism in mediating the compensatory modulation of OGT expression for maintaining *O*-GlcNAc homeostasis in lung cancer cells. However, it is well established that many *O*-GlcNAcylation sites can also serve as phosphorylation sites, and there may exist a yin–yang type of functional interplay or crosstalk between protein *O*-GlcNAcylation and phosphorylation [6]. Although Ser5 and Ser6 of 4E-BP1 have never been previously reported as *O*-GlcNAcylation sites, high-throughput phosphoproteomic analyses have revealed phosphorylation on Ser5 of human 4E-BP1 and on both Ser5 and Ser6 of mouse 4E-BP1 [46,47,48]. Although the functional significance of phosphorylation on these Ser residues has not been investigated, the S5A/S6A 4E-BP1 mutant protein we used may theoretically have a defect in some phosphorylation-mediated function. Therefore, we cannot exclude the possibility that it is the phosphorylation of 4E-BP1 on Ser5 and Ser6 that is important for supporting 4E-BP1 function in regulating OGT translation. Further investigation is necessary to delineate the role of *O*-GlcNAcylation and phosphorylation on these sites.

In conclusion, results of the current study and findings by others together have strengthened the notion that the expression of OGT and OGA is governed by multilayered feedback mechanisms for fine-tuning intracellular *O*-GlcNAc levels to maintain homeostasis. We suggest that OGT is regulated through the control of translational efficiency by 4E-BP1, while the expression of OGA could involve epigenetic mechanisms in transcriptional control in lung cancer cells. Elucidation of the molecular mechanisms underlying *O*-GlcNAc homeostasis will not only reveal how cells respond to fluctuations in *O*-GlcNAc levels but could also uncover possible molecular mechanisms involved in diseases that result from *O*-GlcNAcylation dysregulation.

## 4. Materials and Methods

### 4.1. Cell Culture and Treatments

CL1-5 cells, which were kindly provided by Dr. Pan-Chyr Yang, and A549 cells were maintained in Roswell Park Memorial Institute (RPMI) 1640 medium. Culture media were supplemented with 10% fetal bovine serum and 1% (*v/v*) penicillin/streptomycin. All cells were cultured at 37 °C in a humidified atmosphere with 5% CO_2_. The cells were treated with 5 μM OGA inhibitor Thiamet G (TMG; cat. no. 13237, Cayman Chemical, Ann Abor, MI, USA) or 50 μM PUGNAc (A7229, Sigma-Aldrich, St. Louis, MO, USA), in the complete medium for 24 h to increase *O*-GlcNAc levels. To manipulate the hexosamine biosynthetic pathway (HBP), 10 mM Glucosamine (G1514, Sigma-Aldrich, St. Louis, MO, USA), or 40 μM 6-Diazo-5-oxo-L-norleucine (DON) (D2141, Sigma-Aldrich, St. Louis, MO, USA) were used in the complete medium for 24 h.

### 4.2. Production of shRNA-Expressing Lentiviruses and Infection for Gene Knockdown

Plasmids for the expression of shRNAs targeting OGT (TRCN0000035064, shOGT-1 and TRCN0000035067, shOGT-2), OGA (TRCN0000275512, shOGA-1 and TRCN0000275576, shOGA-2) and 4E-BP1 (TRCN0000040206, sh4E-BP1) were obtained from the National RNAi Core Facility Platform (located at the Institute of Molecular Biology/Genomic Research Center, Academia Sinica, Taipei, Taiwan), which is supported by the National Core Facility Program for Biotechnology, Taiwan. Viral packaging and target cell infection were performed according to the protocol from the National RNAi Core.

### 4.3. Western Blotting Analysis

Cells were lysed in a modified Radio-Immunoprecipitation Assay (RIPA) buffer. Protein samples (50 μg) were subjected to SDS-PAGE and transferred to polyvinylidene fluoride transfer membranes. The membranes were blocked with 5% bovine serum albumin and developed overnight using appropriate primary antibodies at 4 °C. Antibodies used in this study are listed in Table S1 and a detailed procedure is described in the Supplementary Materials. For quantitative analysis, signal intensities from multiple independent experiments were quantified using ImageJ software (NIH, Bethesda, MD, USA).

### 4.4. RNA Extraction and Reverse Transcription-Quantitative Polymerase Chain Reaction (RT-qPCR)

Total RNA was isolated using the TRIzol reagent (Invitrogen, Carlsbad, CA, USA). RNA (5 μg) was reverse-transcribed into cDNA using the oligo-d(T) primer and reverse transcriptase in a 20-μL reaction mixture using the First Strand cDNA Synthesis kit (K1622, Thermo Fisher Scientific, Waltham, MA, USA). RT-qPCR was performed using the Applied Biosystems StepOne™ Real-Time PCR System with fast SYBR green master mix (4385612, Life technologies, Carlsbad, CA, USA). The primer sets used in the PCR are shown in Table S2. *GAPDH* was used as an internal control.

### 4.5. Luciferase Reporter Assay

The pGL3-basic and pGL3-control reporter plasmids (Promega, Madison, WI, USA) were used and pRL-TK (Promega, Madison, WI, USA) was used as the normalization control for transfection efficiencies. Plasmids were transfected into different cells using Lipofectamine LTX with Plus reagent (Invitrogen, Carlsbad, CA, USA) according to the manufacturer’s instructions. Cells were lysed with *Renilla* Luciferase Assay Lysis Buffer (Promega, Madison, WI, USA) at 24-h post-transfection. The luciferase reporter assay was conducted using the Promega reporter system and luciferase activity was detected using a luminometer (TECMAN Infinite M1000).

### 4.6. Ribosome Pulldown Assay

To investigate the translational activity, a ribosome pulldown assay was performed. Briefly, CL1-5 cells treated with or without TMG were lysed and subjected to immunoprecipitation with RPS6 antibodies. The *OGT* mRNA bound with the ribosome was extracted and analyzed by RT-qPCR. A detailed procedure is described in the Supplementary Materials.

### 4.7. Sucrose Gradient Fractionation and Polysome Profiling

To assess translational efficiency, sucrose gradient centrifugation and polysome profiling were performed as described previously [49]. Briefly, CL1-5 cells treated with or without TMG were lysed and loaded onto a 10% to 50% linear sucrose gradient. After centrifugation, each sucrose gradient sample was split into 13 fractions. Total RNA was extracted and the levels of *OGT* mRNA were analyzed by RT-qPCR. The translational efficiency was calculated as the ratio of polyribosome-associated *OGT* mRNAs (fractions no. 9–13) to total mRNA (all fractions). A detailed procedure is described in the Supplementary Materials.

### 4.8. Immunoprecipitation of 4E-BP1 and Liquid Chromatography (LC)-Mass Spectrometry (MS)/MS Analysis

CL1-5 cells were washed with Phosphate Buffered Saline (PBS) buffer twice and lysed in a modified RIPA buffer (50 mM Tris pH 8.0, 150 mM NaCl, 0.1% SDS, 1% NP-40, 1% Triton X-100, 0.5% sodium deoxycholate, with a protease inhibitor cocktail added immediately before use). An amount of 20 μL protein G magnetic beads were incubated with 1 mg lysates and 1 μg anti-4E-BP1 or control IgG antibodies at 4 °C overnight. Immunoprecipitated samples were washed 3 times in lysis buffer, boiled in sample buffer containing 2-mercaptoethanol and subjected to SDS-PAGE and Coomassie blue staining. In-gel protein digestion by trypsin was performed and digested samples were suspended in 0.1% formic acid and analyzed with the nanoACQUITY™ system (Waters, Milford, MA, USA), which was connected to an Orbitrap Elite hybrid mass spectrometer equipped with a nanoelectrospray ionization source (Thermo Scientific, Waltham, MA, USA). Peptides in each sample were separated using a BEH C_18_ column (25 cm × 75 µm, Waters, Milford, MA, USA) with a segmented gradient of 5% to 35% solution B for 210 min at a flow rate of 300 nL/min. The mobile phase was composed of solution A (0.1% formic acid in water) and solution B (0.1% formic acid in acetonitrile). Eluted peptides were ionized with a spray voltage of 1.7kV and introduced into the LTQ-Orbitrap mass spectrometer. Mass spectrometry was conducted in positive ion mode with data-dependent acquisition (isolation width: 2.0 Da). The resolution of full MS was set at 30,000 with a *m/z* range of 400. Peptide mass spectrum data were obtained by full-mass survey scan (*m/z* range: 350–1600). Ten most multiply charged ions (2^+^ and 3^+^) were selected for MS/MS scan by collision-induced dissociation.

For protein identification and quantification, MS raw data were analyzed using the Peaks7.5 Studio software for proteomics (Bioinformatics Solutions, Canada). The search was carried out against the UniProt human protein database (containing 192,901 protein sequences; released in January 2020; http://www.uniprot.org/, accessed on 22 March 2021). The following search parameters were used: parent mass error tolerance, 50 ppm; fragment mass error tolerance, 0.8 Da; enzyme, trypsin; two missed cleavages, oxidation on methionine (+15.99 Da), and carbamidomethylation on cysteine (+57.02 Da) allowed as variable modifications. The average local confidence (ALC) was > 80%. A decoy database was used to calculate the false discovery rate (FDR), which was set at < 0.1%. A protein was identified when at least one unique peptide was matched. Protein quantitation based on the MS spectra was performed with an in-house software [50].

### 4.9. Statistical Analysis

Statistical analyses were performed by two-tailed unpaired Student’s *t*-test. When *p* < 0.05, results were considered to have statistical significance. All the data shown in this study are the mean ± SD from at least three independent experiments.

## Data Availability

Data presented in this report are available on request from the corresponding authors.

## Supplementary Materials

The following are available online at https://www.mdpi.com/article/10.3390/ijms22073463/s1, Figure S1. Changes of *O*-GlcNAc levels and OGT/OGA protein expression in a time course of glucosamine treatment in lung cancer cells, Table S1. Antibodies used in this study, Table S2. Primers used in this study and detailed Experimental Procedures.
