# Supplementary material for: Feedback Regulation of O-GlcNAc Transferase through Translation Control to Maintain Intracellular O-GlcNAc Homeostasis
Source: Int J Mol Sci. 2021 Mar 27;22(7):3463. doi: 10.3390/ijms22073463 (PMC8037101; doi:10.3390/ijms22073463)
Supplement: Supplementary file 1 [file ijms-22-03463-s001.pdf]

Supplementary Materials for

Feedback regulation of *O*-GlcNAc transferase through translation control to maintain intracellular *O*-GlcNAc homeostasis

Chia-Hung Lin [1], Chen-Chung Liao [2,3], Mei-Yu Chen [4,5,6,7,8,9,*] and Teh-Ying Chou [1,8,9,10,11,*]

1 Division of Molecular Pathology, Department of Pathology and Laboratory Medicine, Taipei Veterans General Hospital, Taipei 11217, Taiwan; chiahunglin0222@gmail.com
2 Metabolomics-Proteomics Research Center, National Yang-Ming University, Taipei 11221, Taiwan; ccliao@ym.edu.tw
3 Metabolomics-Proteomics Research Center, National Yang Ming Chiao Tung University, Hsinchu 30010, Taiwan
4 Faculty of Medicine, School of Medicine, National Yang-Ming University, Taipei 11221, Taiwan; meychen@ym.edu.tw
5 Faculty of Medicine, School of Medicine, National Yang Ming Chiao Tung University, Hsinchu 30010, Taiwan
6 Institute of Biochemistry and Molecular Biology, National Yang-Ming University, Taipei 11221, Taiwan
7 Institute of Biochemistry and Molecular Biology, National Yang Ming Chiao Tung University, Hsinchu 30010, Taiwan
8 Cancer Progression Research Center, National Yang-Ming University, Taipei 11221, Taiwan
9 Cancer Progression Research Center, National Yang Ming Chiao Tung University, Hsinchu 30010, Taiwan
10 Institute of Clinical Medicine, National Yang-Ming University, Taipei 11221, Taiwan; tehying@gmail.com
11 Institute of Clinical Medicine, National Yang Ming Chiao Tung University, Hsinchu 30010, Taiwan
* Correspondence: tehying@gmail.com; Tel.: +886-2-2875-7022 (T.-Y.C.); meychen@ym.edu.tw; Tel.: +886-2-2826-7269 (M.-Y.C.)

This file contains:

Supplementary Tables

Table S1. Antibodies used in this study

Table S2. Primers used in this study

Supplementary Figures and legends

Figure S1. Changes of *O*-GlcNAc levels and OGT/OGA protein expression in a time course of glucosamine treatment in lung cancer cells.

Detailed Experimental Procedures

Supplementary Tables

Table S1. Antibodies used in this study

Antigen	Host	Dilution used in Western analysis	Company	Cat. No.
OGT	Rabbit	1:4000	Proteintech	11576-2-AP
OGA	Rabbit	1:2000	Proteintech	14711-1-AP
O-GlcNAc	Mouse	1:2000	BioLegend	MMS-248R
RPS6	Rabbit	1:2000	Novus Biologicals	NB100-1595
4EBP1	Rabbit	1:2000	Cell Signaling	#9452
Ac-H3	Rabbit	1:1000	Upstate	#06-599
Ac-H4	Rabbit	1:1000	Upstate	#06-598
β-Actin	Mouse	1:20000	Novus Biologicals	NB600-501

Table S2. Primers used in this study

Gene	Forward Primer (5′ to 3′)	Reverse Primer (5′ to 3′)
OGT	CTGTGACCTTACAGCCATTTTACATAG	GCTACTTTTCGTGCCAGGAAA
OGA	TCGAGCAAATAGTAGTGTTGTCAGTGT	CGTGACCGCCATTCTTCAA
GAPDH	GGCATCCTGGGCTACACTGA	AGCCCCAGCGTCAAAGGT

Supplementary Figures and legends

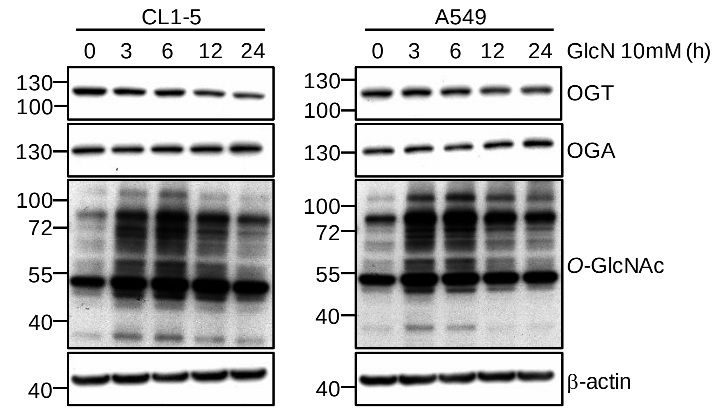

Figure S1. Changes of *O*-GlcNAc levels and OGT/OGA protein expression in a time course of glucosamine treatment in lung cancer cells. Cells were subjected to 10 mM glucosamine (GlcN) treatment and total lysates were prepared at indicated time points. Levels of OGT, OGA and *O*-GlcNAc levels by Western analysis; β-actin was used as a loading control.

Detailed Experimental Procedures

Western analysis

Cells were lysed in a modified RIPA buffer (50 mM Tris pH 8.0, 150 mM NaCl, 0.1% SDS, 1% NP-40, 1% Triton X-100, 0.5% sodium deoxycholate and a protease inhibitor cocktail added immediately before use) on ice for 30 min and centrifuged at 12,000 rpm for 15 min. Protein concentrations were assessed using the BioRad protein assay kit (BioRad). Cell lysates (50 μg proteins) were subjected to 8-12% SDS-PAGE and transferred to polyvinylidene fluoride transfer membrane (PVDF, Pall Corporation). The membranes were blocked for 1 h at room temperature in Tris-buffered saline containing Tween 20 (TBS-T) buffer containing 5% bovine serum albumin (BSA) and then probed overnight at 4°C with a series of primary antibodies. The antibody list for Western blotting was shown in Table S1. β-Actin was used as a loading control. Blots were then incubated for 1 h at room temperature with appropriate horseradish peroxidase HRP-conjugated secondary antibodies and protein signals were visualized by chemiluminescence ECL kit (HyCell International Co., Ltd.).

Ribosome pulldown assay

Briefly, CHX was added to TMG treated CL1-5 cells to a final concentration of 100 μg/mL at 37°C. Five minutes later, cells were washed and harvested in ice-cold phosphate-buffered saline (PBS) with CHX (100 μg/mL). After centrifugation, cell pellet was suspended in lysis buffer (10 mM HEPES, pH 7.9, 100 mM KCl, 5 mM $MgCl_2$, 1% Triton-X 100, 10 U/mL RNaseOUT, 100 μg/mL CHX and 1X protease inhibitor cocktail) on ice for 10 min. The lysates were centrifuged at 8,000 rpm for 10 min and the supernatant was saved as cytoplasmic lysate. About 2 μg of ribosomal protein S6 antibody and Protein G Mag Sepharose were added to 1 mg cytoplasmic lysate and incubated at 4°C overnight. Ribosome complex was washed twice with lysis buffer. The mRNAs bound with ribosome complex were extracted with TRIzol reagent and analyzed the *OGT* mRNA expression level by RT-qPCR.

Sucrose gradient fractionation and polysome profiling

Briefly, CHX was added to CL1-5 cells treated with or without TMG to a final concentration of 100 μg/mL at 37°C. Three minutes later, cells were washed and harvested in ice-cold PBS containing CHX (100 μg/mL). After centrifugation, cell pellet was suspended in RNA lysis buffer (300 mM NaCl, 15 mM $MgCl_2$ $6H_2O$, 15 mM Tris-HCl pH 7.4, 1% Triton-X 100, 100 U/mL RNaseOUT, 100 μg/mL CHX and 1X protease inhibitor cocktail) on ice for 10 min with occasional vortexing. The cell lysates were collected by centrifugation at 12,000 rpm for 10 min at 4°C. The samples were loaded onto linear 10%-50% sucrose gradients and centrifuged at 39,000 rpm at 4°C for 3 h in a SW41 Ti rotor (Beckman). After centrifugation, the gradient fractions were collected on a density gradient fractionator (BR-188, Brandel) and the absorbance was monitored at 254 nm to record the polysome profile. Samples of sucrose gradients were split into 13 subfractions each, starting with 1 (top) to 13 (bottom). Total RNA was isolated using the phenol-chloroform extraction followed by ethanol precipitation. The levels of *OGT* and *GAPDH* mRNAs of individual fraction were quantitated by RT-qPCR. The translational efficiency was calculated in the ratio of polyribosome-associated *OGT* or *GAPDH* mRNAs (9-13 fractions) to total mRNA (all fractions).
